# 

**Published:** 1889-03-16

**Authors:** 


### OUR NEW OFFICES,
### 139 AND 140, SALISBURY COURT, FLEET STREET, E.C.

Our readers are asked to note that with the new volume (which begins on April 6th) portraits of leaders in the medical, philanthropic and nursing world will be issued with the monthly parts. For the convenience of weekly subscribers, these portraits will be issued separately, at 3d. each, but must be ordered at an early date to secure prompt delivery on date of publication. In consequence of the growing requirements of The Hospital, and of the inconvenience to the trade arising from the distance of our present offices from the publishing centre,

**ON AND AFTER APRIL 1st**

The Hospital will be issued from, and all advertising and other communications should be sent to Our New Offices, 139 and 140, Salisbury Court, Fleet Street, E.C.

### Scraps and Gleanings.

Beckenham boasts an excellent Provident Dispensary.

Dartmouth Cottage Hospital treated 36 patients last year.

The smallpox mortality in England last year was 36 per million.

Another £40 has been secured for a free cot in Stockton Hospital.

Besley Cottage Hospital was very economically managed last year.

Woodlands Convalescent Home has begun the year free from debt.

The Peabody Donation Fund built three new blocks of buildings last year.

Kidderminster Infirmary appeals to the workmen for further support.

Teddington Cottage Hospital has reduced its expenditure to 4s. 6d. per day per head.

In the current number of *Blackwood*, Dr. Creighton has an article on Falstaff's Deathbed.

Paisley Convalescent Home admitted over 700 patients last year. It has a deficit of £371.

An amateur operatic performance was given at Kilburn last week in aid of the dispensary.

The Provident branch of Norwich Dispensary is steadily increasing. This is as it should be.

Dr. Lellon, the hero of the Nice elopement, has been sentenced to 8 months' imprisonment.

Lady Clark was present at the *déjeuner* given at the opening of the Medical Session at Haslar.

Dr. E. Houze, of Brussels, says that the tannin treatment of phthisical patients gives good results.

Batley Cottage Hospital being economically managed, can boast a balance in hand of over £200.

Dr. Sydney Martin gave an interesting lecture on "Serpent Poisons" at the Royal Institution last week.

Lord Windsor presided at the annual meeting of Governors of Birmingham Homœopathic Hospital.

Kingston Provident Dispensary has done good work for 23 years. It wants some more honorary members.

Bishop Wilkinson has presented the Waifs and Strays Society with 37 acres of freehold land at Walsham.

The working men of Hull are to be congratulated on the honourable way in which they support the Infirmary.

The Rev. J. G. Wood, the well-known naturalist, lately deceased, was the son of a surgeon of Middlesex Hospital.

Measles are still very bad in many country towns and villages, but in London the epidemic is gradually decreasing.

Beau Site Convalescent Home treated 175 patients in 1888, 150 in 1887, and 36 in 1886. This is steady progress.

The Kent County Ophthalmic Hospital issues a good report: 312 in-patients, 2,225 out-patients; expenditure, £1,441.

The Bishop of Bedford lately preached at St. John's, Hoxton, in aid of the Royal Hospital for Diseases of the Chest.

It is reported that in a Russian village where vaccination was not practised, 1,095 children out of 2,370 took smallpox.

The 400 Glasgow police who have passed their ambulance examination wear a white shield with a red cross on the right sleeve.

The Senatus of the Aberdeen University has resolved to confer the honorary degree of LL.D. on Dr. Lauder Brunton, London.

Dr. W. F. Murray traces the outbreak of enteric fever at Forfar last April to a dairy where the cows were suffering from "stiffness."

More stringent sanitary measures are to be taken in the Spanish ports, in consequence of yellow fever in the Spanish West Indies.

A hospital for fish has been opened at the Midland Counties Fish Culture establishment at Malvern Wells, by Mr. William Burgess.

The people of Marylebone held a meeting last week to protest against the encroachment of the Samaritan Hospital on the Marylebone Road.

Mr. H. Spicer has promised £1,000 to the Samaritan Fund of the London Hospital, on condition that a further sum can be raised of £3,000.

Dr. Galabin in his inaugural address as president of the Obstetrical Society, commented on the present low rate of mortality in our lying-in hospitals.

The Lady Guide Association, of which particulars have from time to time appeared in The Hospital, is being formed with a capital of £5,000, in £1 shares. The registered offices are in Pall Mall.

It is said that Sir Edwin Chadwick, the veteran sanitarian, is about to agitate for the abolition of the tall hat, and the substitution of some headcovering less conspicuous and possibly more serviceable.

Surbiton Cottage Hospital refuses to get into debt, and so for two months only received emergency cases. The inhabitants of Surbiton should appreciate its sacrifice and spirit, and make it unnecessary for such measures to have to be taken again.

A fancy dress ball on behalf of Chelsea Hospital for Women took place on February 27th, at the Princes' Hall, Piccadilly. The rule of strictly fancy dress was rigidly adhered to. Over 250 persons were present, and dancing was kept up with unremitting energy till the small hours of the morning.

An American inventor, Mr. John J. Greenough, has fashioned an ear-piercing apparatus by which the needle is instantly projected through the ear and retracted, the needle also carrying the wire of an earring to remain lodged in the incision, the hole being bored, the earring inserted, and the needle withdrawn at a single instantaneous operation.

At the Liverpool Police-court last week, John Kynaston appeared to answer summonses taken out by the Lunacy Commissioners charging him with receiving and lodging lunatics, his house not being registered or licensed for their reception. Evidence was given as to three persons being sent to the defendant's house suffering from delusions and hysteria. The stipendiary declined to deal with the case and sent it to the assizes.

### Amusements and Relaxation.

### NEW PUZZLE PRIZE SCHEME.

SECOND QUARTERLY COMPETITION COMMENCED JANUARY 5th, ENDS MARCH 30th, 1889.

1. Prizes will be awarded for the best Original Puzzles in Prose or Verse relating to any of the Social, Political, or Philanthropical topics of the day. Competitors are requested to *strictly* observe this rule, or their contributions will be discredited.

2. Puzzles may take any form which the ingenuity of the competitors suggest. Answers must accompany contributions, and the source of obscure or ambiguous terms be given.

3. No competitor will be allowed to take more than one prize during the Quarter.

4. Eight Prizes of Standard Books will be given, the choice of the book to be left to the winner. First, Second, Third, Fourth, and Fifth Prizes for best Original Puzzles, value £1 1s., 15s., 10s. 6d., 7s. 6d., and 5s. each.

5. First, Second, and Third Prize for Solutions of 15s., 10s. 6d., and 5s. each.

N.B.—All contributions must reach the office, 38, Old Jewry, E.C., not later than the *First Post* on *Thursday* in each week.

### PUZZLES FOR SOLUTION.

### Eleventh Week.

No. 23. Double Acrostic.

The birth of my finals in primals takes place,
And all must admire its sweet beauty and grace.

1. Once the sign of glorious conquest,
Given to the victor bold;
Fading, it must be confessed,
Ere his triumphs had grown old.

2. Title, used in Eastern land
To show authority and power;
Bestowed on one in high command,
Or blessed with learning's ample dower.

3. I may be a figure, I may be a word,
But in either a number must show;
Misplaced, I may read as something absurd,
As children very well know.

4. In olden days, the Norman dames
Applied themselves to this;
And there portrayed the noble games
In which their lords found bliss.

Patience.

No. 24. Diamond Puzzle.

.
. . .
. . . . .
. . . . . . .
. . . . .
. . .
.

My first a consonant you see,
My next is famed for industry,
My third when shot flies speedily;
My whole read downwards and along
A country shows both great and strong,
Upon whose doings and intent
The eyes of Europe late were bent.
My fifth may fire a rick of hay,
My sixth doth work and knows no play.
My last—it is the end of day.

Bijou.

### Marks for Original Puzzles.

| Names. | No. of Puzzles sent in Mar. 7. | No. of Marks Mar. 7. | Totals. | Names. | No. of Puzzles sent in Mar. 7. | No. of Marks Mar. 7. | Totals. |
|---|---|---|---|---|---|---|---|
| Cotonopolis... | 1 | 3 | 40 | Prior............ | 3 | 3 | 19 |
| Tinie............ | 3 | 5 | 44 | Lightowlers . | 3 | 4 | 24 |
| Jenny Wren . | — | — | 4 | Twentieth ... | — | — | 3 |
| Patience ...... | 3 | 7 | 53 | Crocodile...... | — | — | 8 |
| Padre ......... | — | — | 3 | Nabob ......... | 1 | 3 | 11 |

### Answers.

No. 19. Double Acrostic.

E vi L
A pocryph A
R ui N
T umeri C
H avann A
Q uietu S
U ta H
A lib I
K ohi-noo R
E rmin E

No. 20. Enigma. Coal.

### Marks for Solution of Puzzles.

Total Marks.—Flora (1), 9; Mater (2), 7; Scrooge (1), 9; Patience 4; Lady Clara (2), 9; Ruffles (0), 6; Lauriston, 1; Reldas (1), 6; Jenny Wren, 4; Condorcet 2; Tinie, 2.

[*The Figures in parentheses are Marks for the week ending March 7th.*]

### Conditions.

I. Every paper sent in must be clearly written, and one side only must be used. All competitors must mark at the head of their papers the number of their competition.

II. Each paper must be signed by the author with his or her real name and address. A *nom de plume* may be added if the writer does not desire to be referred to by us by his real name. In the case of all prizewinners, however, the real name and address will be published.

III. All communications relating to prize competitions should be addressed to "The Prize Editor, The Hospital, 38, Old Jewry, London, E.C." Competitors are requested not to include in letters, etc., to the Prize Editor, communications on matters of other business. All letters must be fully prepaid.

IV. The Prize Editor of The Hospital is the sole judge of the competitions, and his decision is in all cases final and without appeal.

V. The Prize Editor reserves the right to publish any paper sent in, whether it receives a prize or not. He does not hold himself responsible for any MSS. sent, nor can he undertake to return rejected competitions.

